# Preemptive one lung ventilation enhances lung collapse during thoracoscopic surgery: A randomized controlled trial

**DOI:** 10.1111/1759-7714.13091

**Published:** 2019-05-21

**Authors:** Yunxiao Zhang, Wanpu Yan, Zhiyi Fan, Xiaozheng Kang, Hongyu Tan, Hao Fu, Zhendong Li, Ke‐Neng Chen, Jiheng Chen

**Affiliations:** ^1^ Key Laboratory of Carcinogenesis and Translational Research (Ministry of Education/Beijing), Department of Anesthesiology Peking University Cancer Hospital & Institute Beijing China; ^2^ Key Laboratory of Carcinogenesis and Translational Research (Ministry of Education/Beijing), The First Department of Thoracic Surgery Peking University Cancer Hospital & Institute Beijing China

**Keywords:** Lung collapse, one‐lung ventilation (OLV), thoracoscopic surgery

## Abstract

In routine practice, one lung ventilation (OLV) is initiated upon pleural opening. We conducted a randomized controlled trial to compare lung collapse after preemptive OLV versus conventional OLV in thoracoscopic surgery. A total of 67 patients were enrolled (34 with conventional OLV; 33 with preemptive OLV). Preemptive OLV was conducted by closing the DLT lumen to the non‐ventilated lung immediately upon assuming the lateral position with the distal port closed to the atmosphere until pleural opening (>6 minutes in all cases). Lung collapse was assessed at 1, 5, 10, 20, 30 and 40 minutes after pleural opening using a 10‐point rating scale (10: complete collapse). The primary end point was the duration from pleural opening to satisfactory lung collapse (score of 8). Secondary end points included PaO_2_ and hypoxemia. The duration from pleural opening to satisfactory lung collapse was shorter in the preemptive OLV group (9.1 ± 1.2 vs. 14.1 ± 4.7 minutes, *P* < 0.01). PaO_2_ was comparable between the two groups prior to anesthetic induction (T0), and 20 (T2), 40 minutes (T3) after pleural incision, but was lower in the preemptive OLV group at zero minutes after pleural incision (T1) (457.5 ± 19.0 vs. 483.1 ± 18.1 mmHg, *P* < 0.01). No patients in either group developed hypoxemia. In summary, preemptive OLV expedites lung collapse during thoracoscopic surgery with minimal safety concern.

## Introduction

Effective collapse of the non‐ventilated lung facilitates video‐assisted thoracoscopic surgery (VATS).^1^, ^2^ The lung collapse consists of two phases, with distinct mechanisms and contribution to the process of lung collapse.^3^ Phase I occurs immediately after the opening of the non‐ventilated lung to the ambient air as a result of inherent elastic recoil, and lasts until small airways close. Phase II of the lung collapse depends on gaseous diffusion and absorption atelectasis. A variety of methods have been developed to enhance lung collapse during thoracoscopic surgery. Attempts to accelerate phase I lung collapse, including carbon dioxide insufflation or bronchial suction, generally produce minimal impact on the duration to achieve satisfactory lung collapse since Phase I typically lasts only for 60 seconds.^4^, ^5^, ^6^ Pure oxygen has been shown to be superior to the mixture of oxygen and air in speeding phase II lung collapse when used for two lung ventilation before OLV^7^ as it is more easily absorbed into the blood.^8^ Other methods for accelerating lung collapse include ventilation with nitrous oxide^9^ and use of the disconnection technique.^10^, ^11^

A previous study^12^ showed that at an inspired alveolar ventilation/perfusion ratio of 0.001, it requires no more than six minutes for a lung unit to collapse with a fraction of inspired oxygen of 1.0. We therefore speculated that initiating OLV at least six minutes prior to pleural opening to create a confined space to the non‐ventilated lung when inhaling pure oxygen could expedite lung collapse. This can be achieved by clamping the double‐lumen endobronchial tube (DLT) lumen to the non‐ventilated lung at least six minutes earlier, but not opening the distal port of the lumen to the atmosphere until pleural opening, which prevents air entering the non‐ventilated lung due to the tidal movement caused by the ventilated lung.^13^

In this randomized, double‐blinded controlled trial, we investigated whether preemptive OLV could accelerate the collapse of the non‐ventilated lung in patients with lung cancer requiring OLV for VATS.

## Methods

### Study design and patients

This randomized parallel group trial enrolled lung cancer patients scheduled to receive VATS under OLV between December 5, 2017 and March 1, 2018 at Beijing Cancer Hospital, Beijing, China. Major inclusion criteria were patients had to be 18 to 65 years of age, with an American Society of Anesthesiologists (ASA) physical status I or II and a body mass index between 18 and 25 kg/m^2^. Major exclusion criteria were pneumothorax or using artificial penumothorax, abnormal expiratory recoil [forced expiratory volume at 1 second (FEV1) < 70% of predicted value], pleural adhesion anticipated during preoperative assessment, or bullae on chest computed tomography scans, a history of severe asthma, COPD or thoracic surgery, a risk of blood or infected secretions contaminating the dependent lung as well as expected difficult intubation.

The study protocol was approved by the Ethics Committee of Beijing Cancer Hospital (2016YJZ03). Written informed consent was obtained from all participants. The trial was registered at the Chinese Clinical Trial Registry (ChiCTR‐IOR‐17013667).

### Randomization

Eligible patients were randomized to receive conventional or preemptive OLV. The allocation sequence was generated using a computer program by a staff member not otherwise involved in the trial. Concealment was carried out using opaque envelopes that were opened upon patient's arrival at the operating room. The operating surgeon was blinded to patient assignment.

### Anesthesia

Anesthesia induction was achieved with 0.3 μg/kg sufentanyl, 2 mg/kg propofol, and 1 mg/kg of rocuronium after placement of monitors. Intubation was carried out using a left‐sided DLT and the patient was then connected to mechanical ventilation. DLT placement was confirmed by fiberoptic bronchoscopy and adjusted as needed. All patients were ventilated with pure oxygen for at least three minutes for thorough denitrogenation before being turned to the lateral position. Anesthesia was maintained with continuous infusion of propofol 120–200 μg/kg^−1^min^−1^ and rocuronium boluses (0.15–0.3 mg/kg) as deemed necessary. Tidal volume was 6–8 mL/kg ideal bodyweight (male: height ‐100, and female: height ‐ 105) without positive end‐expiratory pressure.

### OLV

In the conventional OLV group, OLV was initiated by clamping the lumen of the DLT to the non‐ventilated lung and opening the distal port of the DLT lumen to the atmosphere immediately prior to pleural opening. In the preemptive OLV group, the lumen of the DLT to the non‐ventilated lung was clamped immediately after the patient was turned to the lateral position while the distal port of the DLT lumen was not opened to the atmosphere until pleural opening. The time between lateral position and pleural opening (T L‐P) was recorded, which was guaranteed to be no less than six minutes. In both groups, the lumen of the DLT to the non‐ventilated lung was clamped at the end of expiration to expect the independent lung so that it contained the minimum amount of gas possible.

The surgeon explored the pleural cavity and assessed the lung collapse score at 1, 5, 10, 20, 30 and 40 minutes after pleural opening using a verbal rating scale from 0 (no lung collapse) up to 10 (complete collapse)^7^ via video view, and the time from pleural opening to satisfactory lung collapse (collapse score of 8) was recorded. When the operation was completed, the patients were discharged to the post‐anesthesia care unit (PACU).

### Outcome measures

Arterial blood gases were obtained while patients were inhaling air before anesthetic induction (T0), and at 0 (T1), 20 (T2), and 40 minutes (T3) after pleural incision. Intraoperative hypoxemia (SpO_2_ < 90%), intraoperative use of continuous positive airway pressure (CPAP) during OLV, the duration of OLV and the duration of surgery were also recorded.

The primary end point was the duration from pleural opening to satisfactory lung collapse (collapse score of 8). Secondary end points included PaO_2_ at T0, T1, T2 and T3, the incidence of intraoperative hypoxemia and intraoperative use of CPAP, as well as the duration of surgery and OLV. Data were recorded by the anesthesiologist using a standardized study case report form and later entered into a computerized database.

### Statistical analysis

In the current study, the primary end point was the duration to satisfactory lung collapse (score of 8) as this degree of lung collapse is sufficient to allow surgery to commence. Since there was no data reporting the duration to collapse with a score of 8, we calculated the sample size based on a previous report of duration to complete lung collapse (score of 10). A power analysis indicated a minimum of 23 subjects are needed in each group based on the following assumptions: (i) α at 0.05; (ii) 1‐β at 0.9; (iii) the primary end point measure (duration to lung collapse score of 10) in the conventional OLV group at 22 ± 3.6 minutes^10^; and (iv) clinically meaningful 3.5‐minutes reduction of the primary end point measure in the preemptive OLV group. Considering possible attrition (due to lung adhesion or intubation failure) and the primary measure of collapse at a score of 8, rather than score of 10, we expanded the sample size calculation by a factor of 1.65, and planned to enroll a total of 76 subjects. The primary end point was analyzed using Student's *t*‐test. Data are expressed as mean ± standard deviation (SD). Categorical variables were analyzed using the chi‐squared‐test. Statistical analyses were carried out using SPSS version 22.0 (SPSS Inc. Chicago, IL, USA). A *P*‐value < 0.05 (2‐sided) was considered statistically significant.

## Results

### Patient demographic and baseline characteristics

**Figure 1:**
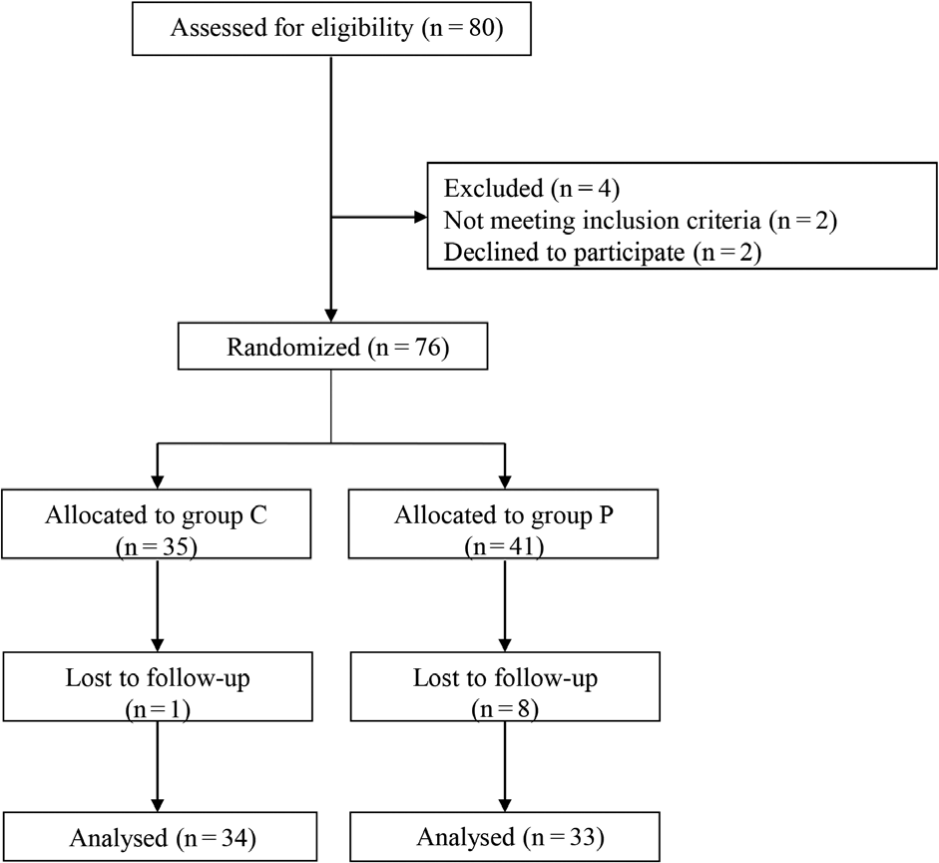
The study flowchart.

The study flowchart is shown in Fig 1. A total of 80 lung cancer patients requiring OLV for VATS were assessed for eligibility. Four patients were excluded because two of them declined to participate in the study and another two patients did not meet the inclusion criteria. Finally, 76 patients were randomized to receive either conventional OLV (n = 35) or preemptive OLV (n = 41). One patient in the conventional OLV group and eight patients in the preemptive OLV group had pleural adhesion and were excluded in the final analysis. The final analysis included 34 patients in the conventional OLV group and 33 patients in the preemptive OLV group. The two groups were comparable in demographic and baseline variables (Table 1).

**Table 1:** Demographic and baseline characteristics

Variable	Preemptive OLV (*n* = 33)	Conventional OLV (*n* = 34)	*P*
Age (years)	55.9 ± 9.8	54.9 ± 5.6	0.610
Sex (M/F)	13/20	14/20	0.540
Weight (kg)	66.2 ± 10.5	67.6 ± 11.2	0.615
Height (cm)	162.6 ± 9.1	168.3 ± 8.3	0.578
ASA class I/II	5/28	4/30	0.480
FEV_1_ /FVC (%)	78.9 ± 5.5	78.8 ± 6.3	0.935
Type of surgery			0.946
R VATS (lobectomy)	16	16	—
R VATS (wedge/segmentectomy)	3	4	—
L VATS (lobectomy)	11	12	—
L VATS (wedge/segmentectomy)	3	2	—
Surgeon			0.981
A	22	22	—
B	9	10	—
C	2	2	—

Data are expressed in mean ± SD or number.

ASA, American Society of Anesthesiologists; FEV_1_/ FVC, forced expiratory volume at 1 second/forced vital capacity (lung function test); VATS, video‐assisted thoracoscopic surgery.

### Primary end point

Duration from pleural incision to satisfactory lung collapse was significantly shorter in the preemptive OLV group (9.1 ± 1.2 vs. 14.1 ± 4.7 minutes in the conventional OLV group, *P* < 0.01) (Table 2). The lung collapse score in the preemptive OLV group was 3.6 ± 0.8 at 1 minute, 5.9 ± 0.8 at 5 minutes, 8.5 ± 0.8 at 10 minutes and 9.4 ± 0.7 at 20 minutes, respectively. At all time points, lung collapse score in the preemptive OLV group was significantly higher than the control (*P* < 0.01 for all) (Table 3).

**Table 2:** The study outcomes

Variables	Preemptive OLV (*n* = 33)	Conventional OLV (*n* = 34)	*P*
Time required for satisfactory lung collapse (minutes)	9.1 ± 1.2	14.1 ± 4.7	< 0.001
Time from lateral position to pleural opening (minutes)	12.0 ± 1.7	11.7 ± 1.7	= 0.454
Intraoperative hypoxemia (n)	0	0	1
Intraoperative use of CPAP during OLV (n)	0	0	1
Duration of surgery (minutes)	106.5 ± 43.0	112.2 ± 42.4	= 0.586
Duration of OLV (minutes)	102.1 ± 32.7	101.1 ± 41.5	= 0.918

Data are expressed in mean ± SD or number.

CPAP, continuous positive airway pressure; OLV, one‐lung ventilation.

**Table 3:** Lung collapse score at selected time points after pleural opening

	1 min	5 min	10 min	20 min	30 min	40 min
Preemptive OLV	3.6 ± 0.8*	5.9 ± 0.8*	8.5 ± 0.8*	9.4 ± 0.7*	9.8 ± 0.4	9.9 ± 0.2
Conventional OLV	1.8 ± 1.0	4.2 ± 1.1	6.7 ± 1.3	8.9 ± 1.1	9.8 ± 0.6	9.9 ± 0.3

* *P* < 0.05 versus the control group.

### Secondary end points

PaO_2_ was comparable between the two groups at T0, T2 and T3, but significantly lower in the preemptive OLV group at T1 (457.5 ± 19.0 vs. 483.1 ± 18.1 mmHg, *P* < 0.01). In both groups, PaO_2_ peaked at T1 and remained significantly elevated at T2 and T3 vs. T0 (*P* < 0.05) (Table 4). No patients in either group developed intraoperative hypoxemia and no patients in either group used CPAP during OLV (Table 2).

**Table 4:** PaO_2_ at selected time points

PaO_2_ mmHg	T0	T1	T2	T3
Preemptive OLV	86.2 ± 7.1	457.5 ± 19.0* ^△^	215.4 ± 28.6*	160.6 ± 27.3*
Conventional OLV	84.9 ± 8.6	483.1 ± 18.1*	217.6 ± 11.0*	162.9 ± 14.9*

* *P* < 0.05 versus T0, ^△^ *P* < 0.05 versus the control group.

Data are expressed as mean ± SD.

The durations of OLV and surgery were comparable between the two groups. The duration from lateral position to pleural opening was also comparable between the two groups (Table 2).

## Discussion

The current study shows that preemptive OLV significantly reduced the time to achieve satisfactory lung collapse, in comparison with conventional OLV (from an average of 14.1 to 9.1 minutes). The result in the control group (14.1 minutes) is consistent with the report in a previous study.^7^ At one minute after pleural incision, the lung collapse score was 3.6 in the preemptive OLV group versus 1.8 in the conventional OLV group, suggesting that the advantage of preemptive OLV starts as early as one minute.

The present study also shows that the preemptive OLV did not cause hypoxemia. No patients in either group developed intraoperative hypoxemia or used CPAP during OLV. Since patients in both groups breathed pure oxygen before pleural incision, PaO_2_ increased sharply after pleural opening (T1) and remained significantly elevated at T2 and T3 compared with T0. This finding is in accordance with a previous study,^7^ in which PaO_2_ became sharply elevated initially, and then showed a steady decline during OLV. The decline of PaO_2_ after OLV may reflect atelectatic regions of the ventilated lung due to gravitational gradient and fall of the mediastinum in the open chest during OLV.^14^ Furthermore, arterial oxygenation in the preemptive OLV group was slightly lower than that the control at T1, possibly due to early OLV.^14^

Despite the early initiation of OLV in the preemptive OLV group, the duration of OLV was not significantly different between the two groups. Also, the duration of surgery did not differ between the two groups. This might reflect the fact that good lung collapse in early OLV from one minute after pleural opening could enable optimal surgical exposure without having to compress the lung parenchyma, facilitate surgical dissection, and reduce the operating time and incidence of postoperative complications.^1^, ^2^

The concept of early OLV has been adopted by many clinicians. However, in routine practice, the distal port of the DLT lumen to the non‐ventilated lung is opened to the atmosphere immediately after being clamped; as a result, air could enter the non‐ventilated lung due to the tidal movement caused by the ventilated lung and delay lung collapse.^13^ Previous studies used the time needed for complete lung collapse as the primary end point^7^, ^10^ whereas the present study uses the duration of time from pleural incision to satisfactory lung collapse (collapse score of 8) as complete lung collapse is not always achievable in every patient.

Limitations of this study include the fact that the surgeons started to operate on the lungs just after pleural incision, which in turn may influence lung collapse. Also, the verbal rating score is rather crude in assessing lung collapse. Alternative methods, such as the distance of lung collapse away from the chest wall, are more subjective, but prone to bias by other factors (such as differing size of the thoraces across patients).

In summary, the current study showed that, in comparison to conventional OLV, preemptive OLV could expedite lung collapse for thoracoscopic surgery with minimal impact on oxygenation during OLV.
